# Ensemble Technique Coupled with Deep Transfer Learning Framework for Automatic Detection of Tuberculosis from Chest X-ray Radiographs

**DOI:** 10.3390/healthcare10112335

**Published:** 2022-11-21

**Authors:** Evans Kotei, Ramkumar Thirunavukarasu

**Affiliations:** School of Information Technology and Engineering, Vellore Institute of Technology, Vellore 632014, India

**Keywords:** tuberculosis detection, deep learning, transfer learning, ensemble learning, lung segmentation, medical image analysis

## Abstract

Tuberculosis (TB) is an infectious disease affecting humans’ lungs and is currently ranked the 13th leading cause of death globally. Due to advancements in technology and the availability of medical datasets, automatic analysis and classification of chest X-rays (CXRs) into TB and non-TB can be a reliable alternative for early TB screening. We propose an automatic TB detection system using advanced deep learning (DL) models. A substantial part of a CXR image is dark, with no relevant information for diagnosis and potentially confusing DL models. In this work, the U-Net model extracts the region of interest from CXRs and the segmented images are fed to the DL models for feature extraction. Eight different convolutional neural networks (CNN) models are employed in our experiments, and their classification performance is compared based on three publicly available CXR datasets. The U-Net model achieves segmentation accuracy of 98.58%, intersection over union (IoU) of 93.10, and a Dice coefficient score of 96.50. Our proposed stacked ensemble algorithm performed better by achieving accuracy, sensitivity, and specificity values of 98.38%, 98.89%, and 98.70%, respectively. Experimental results confirm that segmented lung CXR images with ensemble learning produce a better result than un-segmented lung CXR images.

## 1. Introduction

Tuberculosis (TB) is a contagious disease caused by Mycobacterium, which affects the lungs of humans (pulmonary TB) but can also affect other parts of the body (extrapulmonary TB). In 2019, about 10 million people contracted the disease, out of which 1.4 million died. Tuberculosis was ranked the 13th leading cause of death worldwide and the first single infectious agent ranking above HIV/AIDS [1]. The number of people newly diagnosed with TB in 2020 declined compared to the previous years. The decline was due to COVID-19 protocols, such as wearing face masks and social distancing, among people. The new TB case count was reduced by 18% between 2019 and 2020, from 7.1 million to 5.8 million. On the other hand, the number of people who died from TB increased in 2020 due to the COVID-19 pandemic. In 2020, TB ranked as the second leading cause of death from a single infectious agent after COVID-19 [2]. It is contagious and can spread through sneezing or coughing from infected persons. The most prevalent TB regions are Africa and Southeast Asia, mainly due to limited resources and relatively high poverty rates. TB is most prevalent in South-East Asia (43%), Africa (25%), and the Western Pacific (18%), with smaller shares in the Eastern Mediterranean (8.3%), the Americas (3.0%), and Europe (2.3%) [2].

TB can be combated or eradicated through early detection, based on testing methods such as culture tests, chest radiography, sputum smear microscopy, and nucleic acid amplification. Chest computed tomography, histopathological examination of biopsy samples, and new molecular diagnostic tests can also improve diagnoses [3]. Amongst these tests, sputum smear microscopy and chest X-ray are the most widely used techniques for TB detection [4,5,6,7,8,9]. X-ray is less invasive, making it one of the best medical imaging modalities [10] for diagnosing fractures, luxation, bone and lung disease, location of foreign matters [11] and molecular drug discovery [12,13].

TB comes in many manifestations (infiltrates, consolidation, and cavitation), and diagnosing it through a chest X-ray requires the services of an expert. These experts are not readily available in these high-burden TB countries, hence the need to employ computational techniques to detect the disease early. Artificial intelligence in healthcare delivery has become an essential parameter in the modern healthcare system. Much research has gone into the development of computer-aided diagnosis (CAD) systems [14,15,16,17,18] to augment the decision-making of physicians and radiologists to render effective health care to patients.

This study focused on developing a robust system based on CNN and pre-trained CNNs for automatic TB detection from chest X-ray images. Accordingly, this study proposes a customized CNN model based on global average pooling at the last layer before classification with a support vector machine (SVM) as the classifier. The methodology includes seven pre-trained CNN models for feature extraction and classification based on the X-ray images adapted for the study. The U-Net [19] architecture for image segmentation is utilized to segment X-ray images and produce corresponding lung masks.

The segmented images serve as input data for the customized CNN and the pre-trained models for TB classification. On the other hand, the proposed CNN models were trained on the un-segmented X-ray datasets for TB classification. Training, testing, and validation of the models were based on X-ray images from different populations. This enhances the generalization of the models on an unseen dataset presented to the model for classification. Further, the proposed approach reduces the variance in classification models due to lesser datasets for training. Ensemble learning is employed to handle this drawback and also increase model performance. The outputs from the various individual models are combined to form a CNN ensemble classifier for TB detection. The main contributions of this study are summarized below:

- A robust framework for automatic lung segmentation and TB classification based on chest X-ray images is proposed for the early detection of tuberculosis.
- A combined result from a customized CNN and pre-trained CNN models through stacking ensemble learning is deployed to boost classification accuracy.
- The proposed framework achieved a higher accuracy rate than other state-of-the-art TB detection models, which suggests that our model is better for mass TB screening in regions where TB is much more predominant.

The rest of the paper is structured as follows: Section 1.1 is the background of the study. Section 2 is related to reviewed literature. Section 3 is the proposed methodology and materials employed for the study. Experimental investigations and analysis of results obtained through the proposed approach are presented in Section 4. A detailed discussion of experimental results is conducted in Section 5. Finally, a summary of the study is shown in the Conclusion section.

### 1.1. Background

Deep learning models are widely used in modern healthcare systems. Their implementation ranges from diagnosis, treatment, drug discoveries, precision medicine, and sequence to sequence analysis [16,17,18,19]. Medical image analysis [20,21,22] is a vital area in which deep learning models augment decision-making on patients through feature extraction relating to treatment, drug prescription, and prognosis by physicians. Deep learning’s wide patronage by researchers is due to its ability to extract inherent features from the images, contrary to machine learning models that depend on hand-crafted features. TB is considered one of the leading causes of death globally, and early screening based on automatic DL models is required. Segmentation of infected regions, severity analysis, and many more are carried out using DL models [22,23]. With the presence of computers with high processing powers and big data, state-of-the-art deep learning models produce good results in terms of classification and prediction.

#### 1.1.1. Deep Learning

**Figure 1:**
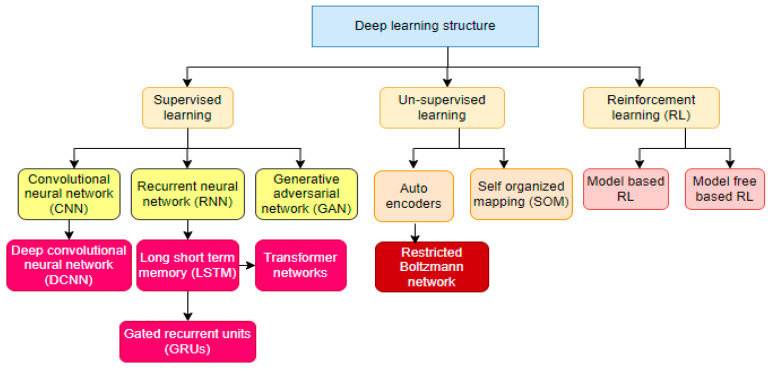
Deep learning algorithms.

Deep learning is a branch of machine learning that involves algorithms, inspired by the structure and function of the brain, called artificial neural networks [24]. Deep learning algorithms have several hidden layers and neurons to extract low-level to high-level features from input data. The lower-level features such as dots, edges, and lines are extracted at the initial layers. High-level features are extracted at the upper layers. A neural network with more than two hidden layers is considered a deep network. In deep learning, extracted features progressively transform through the layers until the output for predictions. Deep learning algorithms proposed in the healthcare domain include:

Convolutional neural networks (CNN) [25], recurrent neural networks (RNN) [26], generative adversarial networks (GAN) [27] and transformer neural networks [28]. Figure 1 depicts deep learning algorithms. CNN is the proven algorithm for computer vision problems such as image processing and medical image analysis. A CNN’s ability to extract low, mid, and high-level feature maps from input data for classification, detection, segmentation, and retrieval tasks makes it superior to other DL algorithms. A CNN is a layer-wise network consisting of an input layer, hidden layers, and an output layer. The hidden layer consists of the convolution layer, pooling layer, non-linear activation function, and fully connected (FC) layer.

**Figure 2:**
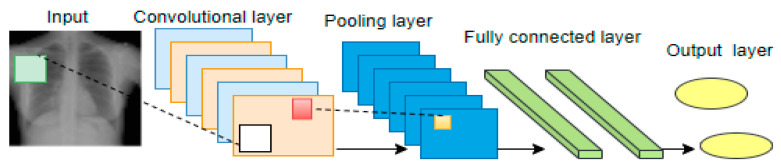
CNN architecture.

A convolutional layer operates on the following characteristics: input and output channels, convolution filters for feature extraction, padding to maintain input dimension at the output layer, and stride for stepping through the input image. The extracted features from the input images are called feature maps. The extracted feature maps from the input layer are passed to the subsequent hidden layers as input until the classification layer. The pooling layer reduces the dimensionality of the feature maps to reduce computational costs. This process reduces the height and width of the feature maps but not the depth. Max pooling and average pooling are examples used in CNN models. The FC layer is the last in the CNN architecture, with a flattened vector built from the output of the preceding layers. A basic CNN architecture is shown in Figure 2.

#### 1.1.2. Support Vector Machine

A support vector machine (SVM) is a leading regression and image classification algorithm for multiple continuous and categorical variables. SVM separates a set of training images into two separate classes, for example, TB positive or TB negative. Given an input, (x1, y1), (x2, y2), …, (xn, yn) where xi in Rd, d-dimensional feature space, and yi in {−1, +1}, the class labels, i = 1 … n, [29]. A hyper-plane is created in the multidimensional space to separate the classes based on a kernel function (K). The SVM classifier is employed to classify images as TB positive or negative because of its performance on binary classification tasks compared to other classifiers.

#### 1.1.3. Deep Transfer Learning

Transfer learning is a machine learning algorithm where a model built for a domain serves as the beginning point for a model on a second domain. It is a general approach in deep learning where pre-trained models form the starting point for computer vision and natural language processing tasks. In situations where the training data is less, an already pre-trained model is engaged, and the knowledge gained in that pre-trained model is transferred to the new task. This process is called transfer learning (TL). Transfer learning’s implementation is in two ways. In the first instance, a pre-trained model is engaged for feature extraction, and the model uses a new classifier that trains on a smaller dataset for classification. In the second instance, the architecture of the adopted pre-trained network is modified to improve the classification performance of the new domain. Mostly the modification happens at the FC layer by replacing a different one with randomly initialized weights, which learn new discriminating patterns from the features.

In [30], the authors used a transfer learning approach based on VGG-16 for COVID-19 detection from chest radiographs. VGG-16 is a pre-trained CNN model with 13 convolutional layers, three fully connected layers, and five max-pooling layers. In their experiment, the last dense layer of the network has two classes (COVID and non-COVID). The second experiment has three categories (COVID, non-COVID pneumonia, and normal) at the output layer. The proposed transfer learning model achieves 96% and 92.5% accuracy in two and three output class cases.

A VGG-16 pre-trained model coupled with an attention mechanism is proposed for COVID-19 detection [31]. The proposed model learns using the COVID-19 CXR image datasets [32,33] for classification. The model’s performance was outstanding compared to other existing methods, making it prudent for COVID-19 screening.

Inception_v3, Xception, ResNet50, VGG19, and VGG16 are utilized in [34] or TB classification. The target dataset serves as input data for the pre-trained models for feature extraction. Training of the target dataset only happens at the classification layer, and the weights at the convolutional layers are frozen and do not contribute to training. Among all the models, Exception, ResNet50, and VGG16 provided the highest classification performance of automated TB classification with precision, sensitivity, F1-score, AUC of 91.0%, and 90.0% accuracy. A further literature review of transfer learning techniques used for TB classification is in Section 2.

#### 1.1.4. U-Net

**Figure 3:**
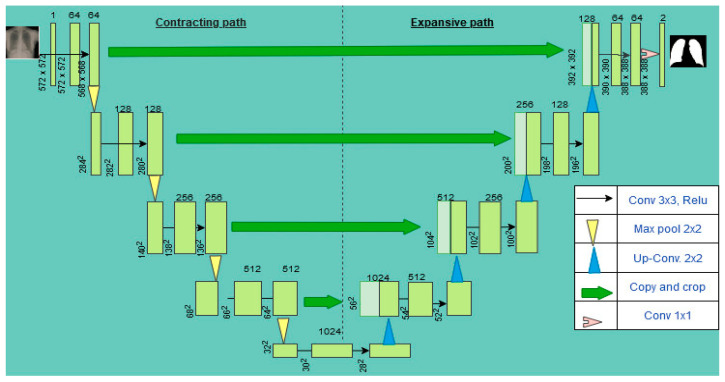
U-Net segmentation and localization network architecture.

U-Net is an architecture purposely designed for biomedical image segmentation and localization tasks in 2015 [19]. Its backbone is the traditional convolutional neural network used for image classification tasks which takes an image as input and produces an output label. U-Net goes further from classification to localization, where an area with an abnormality is detected or localized. It can localize because it classifies every pixel. The architecture is symmetrical and consists of a contracting path (left) and an expansive path (right). The base architecture is shown in Figure 3. The contracting path consists of two 3 × 3 convolutional layers with a rectified linear unit (Relu) and moving channels from 1 to 64 because of an increase in the depth of the image. The input image of size 572 × 572 is reduced to 570 × 570 and then further reduced to 568 × 568. There is a 2 × 2 max pooling with a stride of 2 to halve the size of the input image.

This process gets repeated three times. At the bottom are two convolutional layers without max pooling. Here, the image is resized to 28 × 28 × 1024. The expansive path is a 2 × 2 transposed convolution that up-samples the feature maps by half and concatenates with the corresponding cropped feature map from the contracting path. The final layer is a 1 × 1 convolution that maps each 64-component feature vector to the required number of classes.

## 2. Related Work

**Figure 4:**
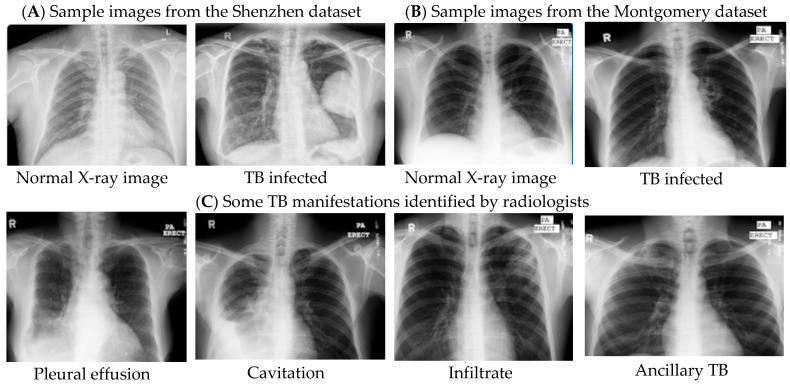
Image samples from both Shenzhen (**A**) and Montgomery (**B**) datasets as well as TB manifestations (**C**).

Computer-aided diagnosis (CADx) [20] is a go-to approach for early screening and automatic detection of TB from chest X-ray radiographs, of which samples are shown in Figure 4 [21]. A typical CAD system consists of three main parts. (i) Data or image segmentation, which is dividing an image into distinct regions, where a region of interest is extracted for analysis [22,23]. (ii) Feature extraction, to produce accurate or exact information such as the shape, texture, and volume of diverse sections of an image. The features are in two classes: geometric features, which extract elements such as points, lines, curves, and surfaces. The other one is appearance features that extract shape-related elements. (iii) Classification methods such as support vector machine (SVM), random forest (RF), and neural networks (NN) have all been used to classify images as normal or diseased [35,36,37].

CAD systems can play a vital role in the analysis of X-ray images for TB detection. It has become possible due to the availability of large-scale labeled datasets, deep learning algorithms, and higher computer graphics processing units (GPUs). In recent years, researchers have shifted the attention from traditional machine learning approaches in developing CAD systems for TB detection [38,39,40,41,42] to deep learning techniques. A convolutional neural network (CNN) is one of the deep learning algorithms that has produced promising results in computer vision tasks. Deep learning is a data-driven technique, but medical images are less in quantity, which poses a threat to effective and robust CAD systems. Knowledge gained from CNN models pre-trained on ImageNet datasets can be transferred through transfer learning to another domain where there is fewer data to learn. Using pre-trained networks to develop CAD systems [43,44,45,46,47,48] produced good results compared to CNN models trained from scratch. A model developed in [49] for TB identification was trained and tested using the Montgomery County chest X-ray (MC) and Shenzhen chest X-ray sets. It achieved an accuracy of 90% and 80%, respectively.

A deep learning-based automatic detection (DLAD) model with a CNN backbone is developed for TB detection based on chest X-ray images [38]. The model had 27 layers with 12 residual connections and operated via a semi-supervised localization approach, as only a fraction of the dataset was annotated. The final layer is split into two, an image-wise classification layer and a lesion-wise localization layer. It recorded sensitivities and specificities for classification, 94.3–100% and 91.1–100% using the high-sensitivity cut-off and 84.1–99.0% and 99.1–100% using the high-septicity cut-off.

**Figure 5:**
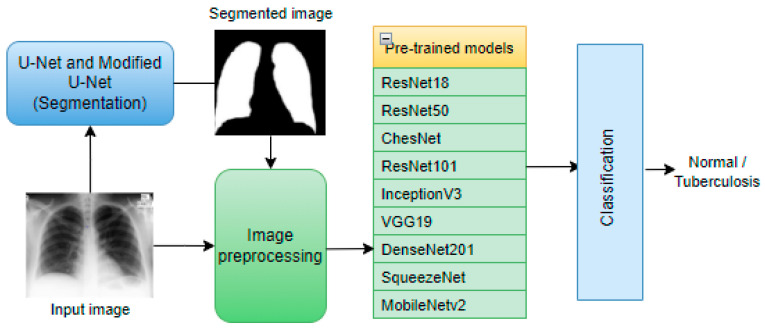
Simplified architecture of proposed framework [53].

Deep convolutional models, such as VGG16 and InceptionV3, combined with a contrast-enhanced canny edge-detected (CEED-Canny) algorithm with an ensemble learning technique are used to classify the images as TB positive or negative [39]. The model achieved accuracy, sensitivity, and specificity values of 93.59%, 92.31%, and 94.87%, respectively. In [50], the authors proposed a CheXNet [51], a deep CNN model, and CNN with an SVM classifier to detect pneumoconiosis from X-ray images. The dataset is from the National Institute for Occupational Safety and Health (NIOSH) [52]. The experimental results showed that the proposed framework was better than other earlier models. SVM performed well, with an accuracy of 92.68%. Despite the success of deep learning models in CAD implementation, it suffers from a problem known as over-fitting. Over-fitting arises as a result of less quantity of data for training a model. Data augmentation is one technique that addresses this problem. In [53], the authors employed data augmentation techniques to detect TB reliably from chest X-ray images. The proposed framework is in Figure 5. Some public databases are combined into one database of 3500 TB infected and 3500 un-infected chest X-ray images for the study.

Already pre-trained networks including ResNet18, ResNet50, ResNet101, ChexNet, InceptionV3, Vgg19, DenseNet201, and SqueezeNet [54,55,56,57,58,59] are adopted for transfer learning. The images are segmented based on U-Net [19] and a modified U-Net architecture. The model achieved accuracy, precision, sensitivity, F1-score, and specificities of DenseNet201 are 98.6%, 98.57%, 98.56%, 98.56%, and 98.54% for the segmented lung images.

The authors in [60] proposed a three-step approach for TB detection from X-ray images. Step (a) modified the CNN model structures, step (b) fine-tuned via an artificial bee colony algorithm, and step (c) implemented a linear average–based ensemble method. The model was trained and validated on the Shenzhen dataset and could segregate seven TB-related manifestations (consolidation, effusion, fibrosis, infiltration, mass, nodule, and pleural thickening). A Bayesian-based convolutional neural network (B-CNN) was deployed in [61] to deal with the SoftMax inference problem. The B-CNN model dealt with model uncertainty well, improving the accuracy to 96.42% and 86.46% for both datasets (i.e., Montgomery and Shenzhen [21]).

To further increase the performance of CNNs in TB detection, a spatial pyramid pooling (SPP) technique was employed [8]. Three pre-trained models, AlexNet, GoogLeNet, and ResNet50, are for feature extraction and classification. GoogLeNet and GoogLeNet-SPP emerged as the best performing models with an accuracy of 97.0% and 98.0%. Deep learning models performed better than traditional machine learning or image processing algorithms. In the study proposed by the authors in [62], TB detection was from computer tomography (CT) images. Four three-dimensional (3D) CNN models, DENSEVOXNET-RPN, 3DUNET-RPN, and VNET-RPN, are trained and evaluated on 501 pulmonary tuberculosis CT images. The model annotated lesions into miliary, infiltrative, caseous, tuberculoma, and cavitary types. Recall and precision detection rates from the model are 98.7% and 93.7%, respectively. A hybrid method [63] for tuberculosis classification was carried out using Shenzhen and Dataset 2 X-ray images datasets. MobileNet and artificial ecosystem-based optimization (AEO) algorithms extract relevant features from the dataset. The algorithm improved the classification performance of the model. The proposed model performed well by attaining an accuracy value of 90.2% and 94.1% for the Shenzhen dataset and Dataset-2, respectively.

Medical images contain sensitive information about the internal organs of a human, aiding doctors in decision-making on the kind of therapy to recommend for a patient. These images captured by different equipment with different resolutions create a problem for CAD systems. To deal with the challenges [64], three image enhancement algorithms called unsharp masking (UM), high-frequency emphasis filtering (HEF), and contrast limited adaptive histogram equalization (CLAHE) are used to boost the quality of the Shenzhen dataset before being trained with EfficientNet and ResNet. The model achieved 89.92% and 94.8% of classification accuracy and AUC (area under curve) scores, respectively.

Ensemble learning is one technique used for TB classification problems. Ensemble learning is a combination of several classifiers to improve the overall classification performance of a model. With this approach, the output from each model is combined to train a new classifier to achieve better accuracy. In the study conducted in [65], an ensemble model with feature descriptors and pre-trained CNN classifier predicted the Shenzhen and Montgomery datasets [21] as containing TB or not. The outcomes from three pre-trained networks, VGGNet, Resnet, and GoogleNet, are combined for further classification [66] using a support vector machine (SVM) classifier. The ensemble classifier achieved accuracy values of 82.6% on the Montgomery dataset and 84.7% on the Shenzhen dataset, respectively.

**Figure 6:**
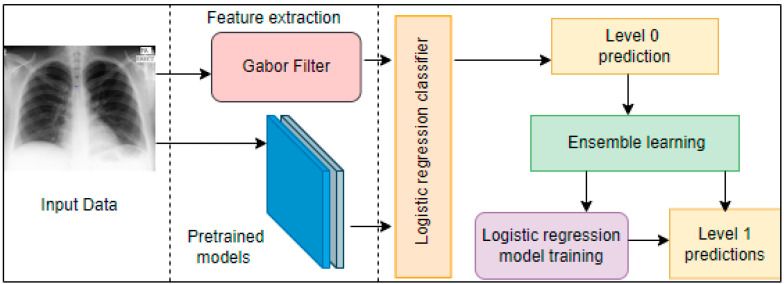
Block diagram of the proposed method [67].

Feature extraction is based on hand-crafted techniques combined with Inception v3, InceptionResnetv2, VGGNet, MobileNet, ResNet50, and Xception, which are employed for feature extraction for TB detection [67]. The experiment was conducted based on the Montgomery and Shenzhen datasets. Predictions from these models are combined for final prediction with a logistic regression classifier through ensemble learning. The proposed model is shown in Figure 6, and it achieved AUC and accuracy scores of 0.99 and 97.59%, respectively.

The above-reviewed related work indicates that deep learning models produce excellent results in TB screening, suggesting that they can be adopted for mass screening, especially in prevalent TB regions. The Montgomery and Shenzhen dataset [21], which is publicly available, is widely adopted by most researchers, even though there were some privately collected datasets. Despite the excellent performances of DL models in TB classification, some limitations have been identified and summarized in Table 1.

## 3. Materials and Methods

**Figure 7:**
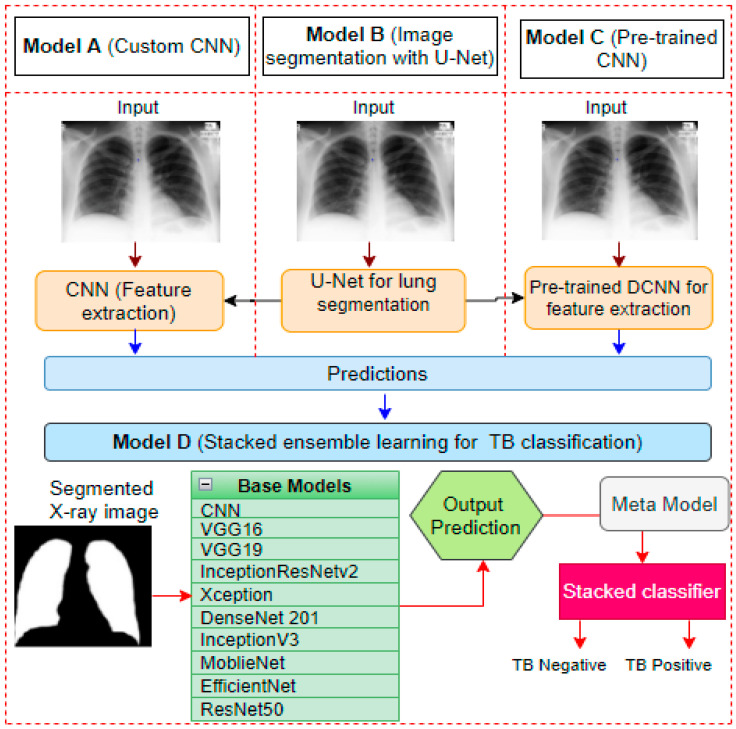
Simplified block diagram of proposed framework.

This section explains the methods and datasets used in the study. Steps such as data pre-processing and feature extraction are used based on supervised learning. Figure 7 illustrates the framework for the proposed system. In this study, we conducted three experiments for enhanced automatic TB detection. In experiment one, the Kaggle dataset consisting of chest X-ray images with corresponding lung masks was segmented with the U-Net model. A new dataset (Chest X-ray Images for Tuberculosis dataset) is fed to the trained U-Net model for segmentation and generation of corresponding lung masks.

Experiment two is the feature extraction and TB classification with our customized CNN and the seven other pre-trained CNN models. This part is also subdivided into two phases. In phase one, un-segmented datasets, Chest X-ray Images for Tuberculosis dataset, Shenzhen dataset, and Montgomery County chest X-ray dataset, from different population sets, are used to train, test, and validate all the CNN models for TB detection. In phase two, the segmented Chest X-ray Images for Tuberculosis dataset produced by the U-Net model are fed as input to both customized CNN and pre-trained CNN models for TB detection. The customized CNN model is developed from scratch, whereas the pretrained CNNs are already trained models with the ImageNet dataset. Training the models with datasets from different populations makes the model generalize well on unseen data. Before training, data preprocessing is performed to increase the quality of the dataset. The input dimensions of the dataset were reduced to enhance the computations by reducing the amount of processing power needed. Rescaling was also applied to keep the pixels in the range of 0 and 1. The third experiment used ensemble learning, which combines the results from all the pre-trained models and the customized CNN model for final classification.

### 3.1. Dataset

In this paper, three different chest X-ray datasets that are publicly available are considered for the experiments. The first is the National Library of Medicine (NLM) dataset, which consists of a pair of datasets (the Montgomery County chest X-ray dataset (MC) and the Shenzhen dataset (SZ) [21]. The Montgomery dataset was collected by the Department of Health and Human Services, Montgomery County, Maryland, USA. The dataset consists of 138 frontal chest X-rays, of which 80 are typical cases and 58 with TB manifestations. The image size is either 4020 × 4892 or 4892 × 4020 pixels. The Shenzhen dataset was collected by Shenzhen No.3 People’s Hospital, Guangdong Medical College, Shenzhen, China. The dataset contains 662 frontal chest X-rays, of which 326 are un-infected, whereas 336 are TB-infected. The sizes can vary but are approximately 3000 × 3000 pixels. The second dataset (Chest X-ray Images for Tuberculosis) was obtained from the Kaggle website [70] with the help of researchers from Qatar University, Doha, Qatar, and the University of Dhaka, Bangladesh, and other collaborators from Malaysia. Chest X-ray images consisting of 800 TB negative and 700 TB positive images are used in this study. The third dataset is the Kaggle dataset, which has 704 chest images with corresponding masks [68].

### 3.2. Image Pre-Processing

The datasets used in this work are frontal chest X-ray images which also contain other regions outside of the lungs and interfere with the detection of TB. These regions can harm the performance of the model. The U-Net architecture was adopted to segment the images to eliminate external features. Resizing the input data was needed since all the adopted pretrained networks had different dimensions. With U-Net architecture, the default input size is 256 × 256 pixels. The input sizes for the CNN models are in Table 2.

### 3.3. Lung Segmentation

The U-net architecture for medical image segmentation is employed for this study. U-Net was selected based on its efficiency and robustness in segmenting medical images.

**Figure 8:**
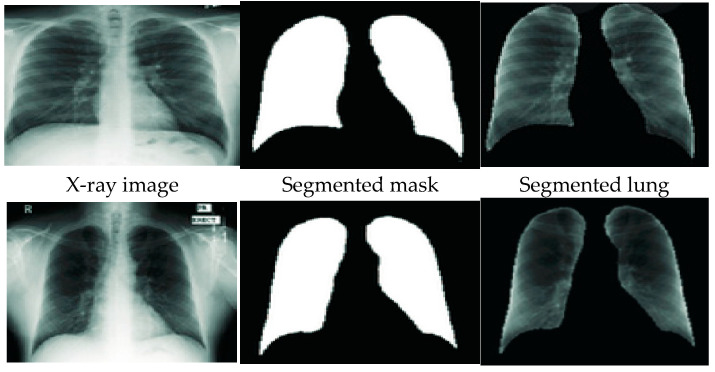
Sample of segmented mask and segmented lung from the base X-ray images.

U-Net consists of two parts, the contracting path, and an expanding path. The contracting or encoding path has two 3 × 3 unpadded convolutions, followed by Relu and 2 × 2 max-pooling layers with stride 2 for down-sampling. The expanding or decoding path contains a 2 × 2 convolution to up-sample the feature maps from the encoder part of the network. The number of features is split and joined with a cropped feature map from the encoder path having two 3 × 3 convolutions and a Relu. Figure 8 shows the samples of the original X-ray image, segmented mask, and the segmented lung generated from the U-Net model. The Kaggle dataset of 704 chest X-ray images, with their corresponding lung masks, was used to train the U-Net model. The dataset is split into 70% training, 20% testing, and 10% validation. The experiment was carried out in the cloud using the Google Colaboratory platform with 12GB NVIDIA Tesla K80 GPU, TensorFlow with Keras library, and Python programming language. The training epoch is set at 30 using Adam optimizer and Dice loss with a batch size of 32.

### 3.4. Feature Extraction Based on CNN and Pre-Trained CNN Models

**Figure 9:**
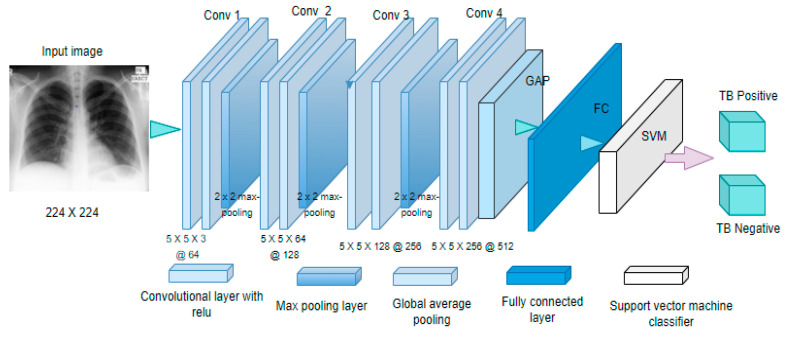
The architecture of the customized CNN model.

The customized CNN model has four convolution layers, shown in Figure 9. Each convolutional layer followed a batch normalization layer, a pooling layer, a Relu activation function, and a dropout layer. Zero padding is performed on the input image to maintain the input dimensions even after classification. The kernel size at the first convolutional layer was kept at 64 and later increased by a scaler of 2 in the preceding convolutional layers. A (5 × 5) kernel was used to convolve with the input image for feature extraction. Batch normalization is then applied to each layer to avoid a common problem in deep learning and ensure that the model generalizes well on unseen data.

The dimensionality of the feature maps generated by the kernels is reduced by spatially averaging the feature maps to a single feature map using global average pooling (GAP). The global average pooling served as an intermediary between the last convolution layer and the fully connected layer, with the Adam optimizer minimizing the categorical cross-entropic loss. The output of the GAP layer is fed to the support vector machine (SVM) for classification. With the help of the ImageNet dataset, seven different pre-trained CNN models extract features from the X-ray images for classification. The weights or parameters in the pre-trained models are kept constant without being trained again to save time and computation power during model training. The extracted features are passed to a SoftMax activation function that classifies an image as clean or TB-infected. The performance of each feature pre-trained model is evaluated based on the individual classifier output.

### 3.5. Classification

Nine pre-trained CNN and customized CNN models are developed for automatic TB detection. Training, testing, and validation datasets are from three databases. The dataset used for classification was in three folds (training, validation, and testing). Data augmentation techniques such as rotation, rescale, shear, zooming, width and height shift, and horizontal flipping helped deal with over-fitting and better generalization. The classification is in two phases. In the first phase, the customized CNN and the pretrained CNNs were trained, validated, and tested on the un-segmented chest images.

Two categories of data, segmented and un-segmented chest X-rays from the chest X-ray images for the tuberculosis dataset, are used to train the models. The feature maps extracted with our customized CNN model are classified as TB positive or negative using the support vector machine (SVM). On the other hand, the SoftMax activation function classifies features extracted by the pretrained models as TB positive or negative.

The outputs from the pretrained models are combined for second-level classification based on the stack ensemble algorithm, [Algorithm 1]. With this algorithm, a meta-model learns how to best combine predictions from all the ten CNN models (base models) proposed in this study. The layers in the base models are frozen to prevent parameter updates when training the stack ensemble classifier for prediction. The predictions from the base models are interpreted to the meta-model with a CNN classifier for final predictions. The proposed stacked ensemble algorithm is below:
**Algorithm 1.** Stack EnsembleInput: Segmented images *(*∝*) = {(x_i,_ y_i_) | x_i_*
∈
*X, y_i_*
∈Y*}* Output: Ensemble classifier *(Ec)*        Step 1: Train base - models *(*φ*)* from segmented images *(*∝*)*        For *p* ← 1 *P* do                Train base model φ base on ∝                Aggregate obtained predictions from all 8 base models        Step 2: Create a new dataset *(*β*)* from base model predictions.        For *n* ← 1 to *z* do                Create new dataset comprising *{x_i,_ y_i_}, where x_i_ = {*β*_j_ (x_i_) for j = 1 − 8}*        Step 3: Train a second-level meta learner                Learn a new classifier *Ec* based on newly created dataset        Return *Es(x) = es(es_1_(x), es_2_(x)… es_8_ (x))*

Table 3 depicts the breakdown of all datasets used in this study. All ten models are implemented in the cloud using the Google Colaboratory platform with 12GB NVIDIA Tesla K80 GPU, TensorFlow with Keras library, and Python 3.7. Each model was trained for 20 epochs. The final classification outputs from the individual models are combined through ensemble learning for better accuracy.

## 4. Results and Analysis

This study proposes a robust system for automatic TB detection from three publicly accessible chest X-ray image datasets using deep learning models. The datasets are of different populations, to train, validate and test the model. In the first step, a U-Net model was trained on the Kaggle dataset for lung segmentation. The metrics below evaluated the performance of the segmentation model.

(1) $$IoU=\frac{\left(TP\right)}{\left(TP+FN+FP\right)}$$

(2) $$Dice\;Coefficient\;\left(F-Score\right)=\frac{\left(2\times TP\right)}{\left(2\times TP+FN+FP\right)}$$

**Figure 10:**
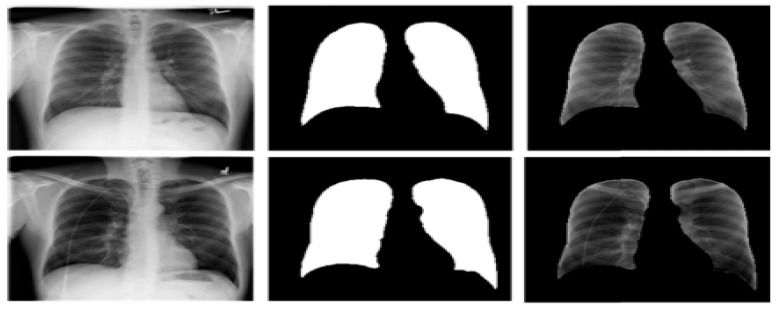
Sample of unseen data with generated mask and segmented lung.

The model achieved a segmentation accuracy of 98.58%, an intersection over union (IoU) of 93.10, and a Dice coefficient score of 96.50. The already trained U-Net model was then used to segment the chest X-ray images shown in Figure 10. The dataset included 800 uninfected images and 700 TB infected images. This was done to prove the robustness of the segmentation model with unseen data. The segmentation model performed very well on the unseen dataset by achieving a segmentation accuracy of 97.99%, IoU of 91.78, and a Dice coefficient score of 95.89.

After segmentation, a qualitative evaluation was performed on the generated mask and the corresponding segmented lung since there was no ground truth mask available in the dataset. This was done to determine how correctly the model had segmented the images. In the second experiment, the customized CNN model and the seven other pre-trained CNN models were trained on the segmented and un-segmented images for TB identification. The chest X-ray images for the tuberculosis dataset were for training, the Shenzhen dataset for testing, and the Montgomery County dataset for validation. Training parameters were set: batch size = 32, learning rate = 0.001, Adam optimizer, training epoch = 20. The proposed model’s performance was evaluated based on accuracy, sensitivity, and specificity. The evaluation metrics for this study are below. TP, TN, FP, and FN denote true positive, true negative, and false positive.

(3) $$Accuracy=\frac{\left(TP+TN\right)}{\left(TP+FN\right)+\left(FP+TN\right)}$$

(4) $$Sensitivity=\frac{\left(TP\right)}{\left(TP+FN\right)}$$

(5) $$Specificity=\frac{\left(TN\right)}{\left(TN+FP\right)}$$

It is important to note that all the pre-trained CNNs and the customized CNN models have different architectures and parameters that cause variation in the results. The results obtained from the individual trained networks on X-ray images without segmentation indicate that VGG19 performed better for classifying the X-ray images by achieving accuracy, sensitivity, and specificity values of 92.86%, 92.86%, and 92.70%, respectively. MobileNet had the lowest sensitivity value of 90% the customized CNN model had the lowest accuracy value of 90.04%. The evaluation results are in Table 4.

The second classification was with the segmented Chest X-ray Images for Tuberculosis dataset. The obtained results increased across all the models compared to the results from the un-segmented images. Even though the performance of all the models increased, the VGG19 model outperformed the remaining models in the TB classification. The VGG19 network achieved accuracy, sensitivity, and specificity values of 97.02%, 97.14%, and 97.14%, respectively.

The customized CNN model had the lowest accuracy values compared to the other models used in this study. Despite the low performance of the CNN model, compared to other existing deep learning models [46], our model performed better by achieving an accuracy of 93.78% on the segmented Chest X-ray Images for Tuberculosis dataset. The obtained results are in Table 5.

In the final study, our proposed stacking ensemble learning technique enhances classification performance. The stack meta-model was trained on the predictions from the individual base models obtained from the segmented lung images. The stacking ensemble performed well because the meta-model learned to correct the variance of the base models by differentially weighing their predictions to produce the best predictions compared to the base models. The stacking ensemble method achieved a maximum accuracy of 98.38%. Classification results obtained from different models for TB detection using segmented images are compared in Table 5.

## 5. Discussion

In this study, the customized CNN model developed for TB detection from datasets of different populations produced a good result. The use of hyperparameter optimization, regularization techniques, batch normalization, and dropout ensured better generalization. On the other hand, all the pre-trained CNN models used in this study performed better than the customized CNN with random weight initializations. Image segmentation was performed on the chest X-ray dataset to exclude unwanted parts in the images with the U-Net model. This was to test the robustness of the segmentation on new datasets. The model achieved an accuracy of 97.99%, an IoU of 91.78, and a Dice coefficient score of 95.89% on the new dataset. The segmented lung images served as input data for classification for both the pre-trained CNNs and the customized CNN models for TB classification. The proposed method is evaluated on three standard metrics accuracy, sensitivity, and specificity. The accuracy values increased across all the models signifying that image segmentation enhances classification. The pre-trained models again performed better than the customized CNN due to the limited dataset for training and testing.

This confirms that deep learning models require a large amount of data to achieve better and acceptable results. Even though all the CNN models performed better in TB classification on segmented and un-segmented images, VGG19 was shown to be the most outstanding model, followed by DenseNet201 in TB detection. VGG19 achieved accuracy, sensitivity, and specificity values of 97.02%, 97.14%, and 97.14% on the segmented chest X-ray images. On the other hand, DenseNet201 achieved accuracy, sensitivity, and specificity values of 96.43%, 95.71%, and 96.57% on the segmented chest X-ray images.

**Figure 11:**
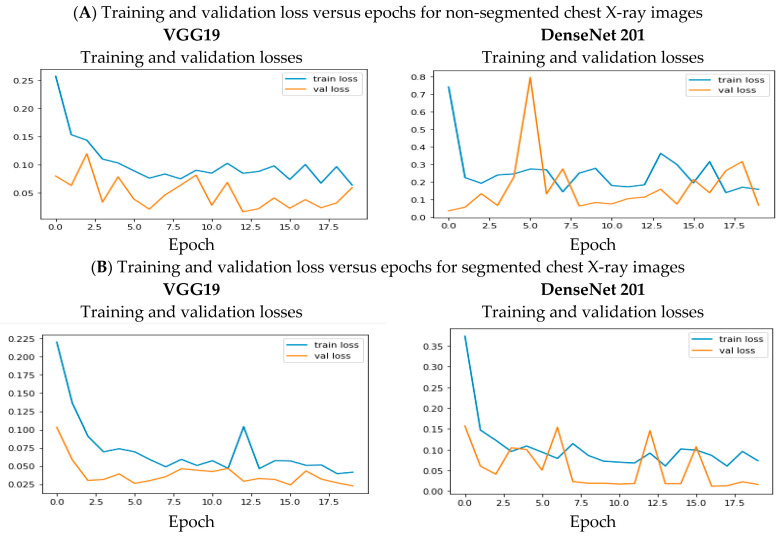
Training and validation losses versus epochs for non-segmented (**A**) and segmented chest e-ray images (**B**).

The loss values for training and validation on segmented images are low compared to un-segmented images. The time to train the segmented images also increased compared to un-segmented images. The training and validation loss obtained for these two outstanding models on both segmented and non-segmented chest X-ray images are in Figure 11. From the training and validation loss curves, some variances are identified and resolved through ensemble learning. All the outcomes from the pre-trained CNN and customized CNN models are combined for further classification using the stacked ensemble classifier.

Different kinds of ensemble techniques, such as majority voting, simple averaging, weighted averaging, and logistic regression, are proposed for TB detection. The stacking ensemble algorithm is adopted for this study because it has a meta-learner, which learns to combine predictions from base models. The output predictions from the base models serve as input, and a meta-learner combines the predictions of the base models for the final prediction. Our proposed stacked ensemble algorithm performed well compared to the logistic regression ensemble technique proposed in [67]. The stacking ensemble learning reduced models’ prediction variance and ambiguity by combining the predictions and delivering optimum performance. The best result in our proposed method was obtained from lung-segmented images using a stacking ensemble classifier. The ensemble classifier achieved an accuracy score of 98.38%. The importance of lung segmentation gave the model cutting-edge over other state-of-the-art models in automatic TB detection from X-ray images. Our proposed methodology confirmed a substantial improvement in automatic TB detection from chest X-ray images by comparing our results to other existing works.

In summary, all the pre-trained CNN models performed well on both segmented images, which indicates that segmented images increase the performance of the deep CNN model compared to the customized CNN. The ensemble classifier achieved the highest accuracy value for TB classification despite being a computationally expensive technique. The performance results obtained from some recently proposed CAD systems for automatic TB detection are compared with the proposed method in Table 6.

## 6. Conclusions

A supervised deep learning model trained on a dataset from one population may not always have the same detection performance when presented with data from another population set. This paper presents a robust deep learning system based on a heterogeneous dataset for automatic TB screening using frontal chest X-ray radiographs. Tuberculosis manifests in many ways, hence the need for a model to automatically classify an X-ray image as TB positive or negative. A U-Net model was used to segment chest X-ray images, which served as input data for our customized CNN and pre-trained CNN models for feature extraction through transfer learning. Through segmentation, classification accuracy improved across all models compared to classification values from un-segmented images. Variance in the output values of the individual models was reduced through stacking ensemble learning. The outcomes from these eight models combined through stacking ensemble learning achieved an accuracy value of 98.38%. This state-of-the-art performance suggests that our proposed model can be used for mass TB screening, especially in areas where TB is much more prevalent. The performance of DL models largely depends on big data.

### Future Work

The performance of DL models depends on a larger dataset, which is not the case with medical images affecting the performance of DL models in a real-time scenario. The performance and robustness of the proposed work can improve by evaluating it on a larger dataset. New augmentation techniques can be developed to introduce more diversity in the dataset to avoid model overfitting. Supervised learning depends on a labeled dataset, which is an expensive and time-consuming task. We recommend that future works focus on unsupervised learning approaches capable of self-generating labels for unlabeled medical datasets for classification. Finally, we recommend the implementation of transformer networks with a self-attention mechanism for visual tasks such as TB detection from X-ray images instead of the already-known CNN models.

## Figures and Tables

**Table 1:** Summary of deep learning methods proposed in literature for TB classification.

Reference	Module	Dataset	Classification	Highlights	Limitations
Msonda et al. [8]	CNN, AlexNet, GoogLeNet ResNet50	Konya Education and Research Hospital, Turkey dataset (Private). Montgomery and Shenzhen dataset [21]	SVM CNN classifier	Presents a methodology that utilizes the DCNN in classifying TB-affected patients using Chest X-Rays (CXR)	Less training dataset which affects model performance
Akbar et al. [49]	CNN	Montgomery and Shenzhen [21]	CNN classifier	TB classification from chest X-ray images based on the CNN model	Less training dataset, and with no data augmentation
Abideen et al. [61]	AlexNet, VGG16, VGG19, CNN	Montgomery and Shenzhen datasets [21]	SVM	Bayesian-based CNN for uncertain cases with low discernibility among TB and non-TB manifested CXRs.	Evaluation performance is less due to less training dataset.
Hooda et al. [38]	CNN	Montgomery and Shenzhen datasets [21]	CNN classifier	Pure CNN model for TB classification	Less training dataset and further verification through a clinical study
Hwang et al. [39]	CNN	Datasets from Seoul National University (Private) Hospital. Montgomery and Shenzhen datasets	CNN classifier	DL–based algorithm for active pulmonary TB detection with lesion-wise localization and image-wise information for detection.	There is uncertainty about the model’s ability to identify different TB manifestations and other pulmonary abnormalities.
Rahman et al. [53]	ResNet, ChexNet, InceptionV3, DenseNet201, Vgg19 SqueezeNet, MobileNet	Kaggle Chest X-ray images [68], RSNA CXR dataset [69]. Montgomery and Shenzhen datasets [21]	CNN classifier	DL model for TB classification and visualization of learning features based on class activation map (CAM) score.	The CAM score shows that the model sometimes learns from the wrong parts of the image, which affects prediction accuracy.
Guo et al. [60]	CNN	Shenzhen datasets [21]	Linear average-based ensemble classifier	Proposed CNN models for TB classification and localization from CXR images	The model can bias due to uneven distribution in class datasets.
Rajaraman et al. [65]	AlexNet, GoogLeNet, VGG-16 and ResNet-50	Indiana School of Medicine and Academic Model Providing Access to Healthcare (AMPATH) dataset. Montgomery and Shenzhen datasets [21]	SVM ensemble classifier	Developed a CNN model through ensemble learning that classifies hand-engineered features for TB detection from X-ray images.	The proposed model is not computationally efficient but rather memory intensive.
Ayaz et al. [67]	Gabor filter, Inceptionv3, VGG-Net MobileNet, ResNet50, Xception	Montgomery and Shenzhen datasets [21]	Linear regression ensemble classifier	TB detection technique that combines hand-crafted features with DCNN through ensemble learning.	The training dataset is less, and the model needs to be evaluated on a larger dataset.
Munadi et al. [64]	ResNet and EfficientNet	Shenzhen datasets [21]	CNN classifier	Presents three image enhancement algorithms for TB detection based on pre-trained CNN models.	The model’s accuracy can be improved since some earlier with a larger dataset.
Li et al. [62]	CNN, 3D DENSEVOXNET-RPN, 3DUNET-RPN	Private dataset	CNN classifier	Developed DL model to diagnose pulmonary tuberculosis (PTB)	Only five types of pulmonary lesions were considered for the study while ignoring the other signs.
Sahol et al. [63]	MobileNet	Montgomery and Shenzhen datasets [21]	K-Nearest Neighbor (KNN)	Proposed a hybrid method based on MobileNet and artificial ecosystem-based optimization (AEO) algorithm for TB classification.	A larger dataset is required for the evaluation of the proposed model
Devnath et al. [50]	CNN	NIOSH dataset [41]	CNN classifier	CNN model for TB classification	A larger dataset is required for further evaluation.

**Table 2:** Parameters and input size of adopted pre-trained CNN networks.

Pretrained Model	Input Size	No. of Parameters	Trainable Parameters	Non-Trainable Parameters
VGG 16	224 × 224	14,764,866	50,178	14,714,688
VGG19	224 × 224	20,074,562	50,178	20,024,384
InceptionResnetv2	299 × 299	54,413,538	76,802	54,336,736
MobileNet	224 × 224	3,090,434	94,082	2,996,352
Xception	299 × 299	21,271,082	409,602	20,861,480
DenseNet201	224 × 224	18,510,146	188,162	18,321,984
InceptionV3	299 × 299	21,905,186	102,402	21,802,784
EfficientNetB1	224 × 224	6,700,681	125,442	6,575,239
ResNet50	224 × 224	23,788,418	200,706	23,587,712

**Table 3:** Un-segmented chest X-ray images from different populations for training, validation and testing.

**Training** **(Chest X-ray Images for TB Dataset)**	**Total No. of Images**	**Normal Images**	**TB Infected**
	1500	800	700
Validation (Montgomery dataset)	138	80	58
Testing (Shenzhen dataset)	662	326	336

**Table 4:** Performance metrics obtained based on un-segmented X-ray images.

Model	Accuracy	Sensitivity	Specificity
Customized CNN	90.04	91.03	90.01
VGG 16	92.38	91.42	92.57
VGG19	92.86	92.86	92.7
InceptionResnetv2	92.62	90.02	93.14
MobileNet	92.3	90	92.71
Xception	91.04	92.14	90
DenseNet	92.38	92.57	91.42
InceptionV3	91.67	92.86	91.43
EfficientNetB1	91.93	91.98	91.67
ResNet50	91.58	91	91.03

**Table 5:** Performance metrics based on segmented X-ray images.

Model	Accuracy	Sensitivity	Specificity
Customized CNN	93.78	91.2	92.24
VGG 16	96.43	95.96	96.71
VGG19	97.02	97.14	97.14
InceptionResnetV2	96.55	96.43	96.57
MobileNet	95.36	95.57	93.01
Xception	95.95	95	96.61
Densenet	96.43	95.71	96.57
EfficientNetB1	96.3	95.21	95.2
ResNet50	95.3	95.5	95.3
InceptionV3	95.76	95.14	92.81
**Ensemble Technique**	**98.38**	**98.89**	**98.7**

**Table 6:** Performance comparison of different computer-aided diagnostic systems proposed for TB classification.

Reference	Feature Extraction	Training and Evaluation Data	Classifier	Evaluation Metrics (%)
Msonda et al. [8]	AlexNet, GoogLeNet ResNet50, CNN	Konya Education and Research Hospital, Turkey dataset. Montgomery and Shenzhen dataset [21]	SVM CNN classifier	Acc = 98, Sens = 97 Spec = 99
Akbar et al. [49]	CNN	Montgomery and Shenzhen [21]	CNN classifier	Acc = 92
Abideen et al. [61]	AlexNet, VGG16, VGG19, CNN	Montgomery and Shenzhen datasets [21]	SVM	Acc = 96.42
Hooda et al. [38]	CNN	Montgomery and Shenzhen datasets	CNN classifier	Acc = 94.73
Hwang et al. [39]	CNN	Datasets from Seoul National University Hospital. Montgomery and Shenzhen datasets	CNN classifier	Acc = 97.7, Sen = 98 AUC = 98.8
Rahman et al. [53]	ResNet, ChexNet, InceptionV3, DenseNet201, Vgg19 SqueezeNet, MobileNet	Kaggle Chest X-ray images [68], RSNA CXR dataset [69]. Montgomery and Shenzhen datasets [21]	CNN classifier	Acc = 98.6 Sen = 98.56 Spec = 98.54
Guo et al. [60]	CNN	Shenzhen datasets [21]	Linear average ensemble classifier	Acc = 98.46 Sens = 98.76 AUC = 99
Rajaraman et al. [65]	AlexNet, GoogLeNet, VGG-16 and ResNet-50	Dataset from Indiana School of Medicine and Academic Model Providing Access to Healthcare (AMPATH). Montgomery and Shenzhen datasets	SVM ensemble classifier	Acc = 96.0 AUC = 96.5
Ayaz et al., [67]	Gabor filter, Inceptionv3, VGG-Net MobileNet, ResNet50, Xception	Montgomery and Shenzhen datasets	Linear regression ensemble classifier	AUC = 99
Munadi et al. [64]	ResNet and EfficientNet	Shenzhen datasets	CNN classifier	Acc = 89.92 AUC = 94.8
Li et al. [62]	CNN, 3D DENSEVOXNET-RPN, 3DUNET-RPN	Private dataset	CNN classifier	Recall = 98.7 Prec = 93.7
Sahol et al. [63]	MobileNet	Montgomery and Shenzhen datasets	K-nearest neighbor (KNN)	Acc = 94.1
Devnath et al. [50]	CNN	NIOSH dataset [41]	CNN classifier	Acc = 87.29
**Prposed model**	CNN, VGG, InceptionResnetV2, MobileNet, Xception, DenseNet, InceptionV3	Chest X-ray images for tuberculosis [70] Kaggle dataset [68] Montgomery and Shenzhen datasets [21]	Stacked ensemble classifier	**Acc = 98.38** **Sen = 98.89** **Spec = 98.70**

## Data Availability

The dataset can be accessed via: Kaggle. Tuberculosis (TB) Chest X-ray Database. Available online: https://www.kaggle.com/datasets/tawsifurrahman/tuberculosis-tb-chest-xray-dataset (accessed on 14 February 2022). Lu, P.X. Chest X-Ray Masks and Label. Kaggle. Available online: https://www.kaggle.com/nikhilpandey360/chest-xray-masks-and-labels (accessed on 20 April 2022).

## References

[1] World Health Organization (2020). Global Tuberculosis Report.

[2] World Health Organization (2021). Global Tuberculosis Report.

[3] Ryu Y.J. (2015). Diagnosis of pulmonary tuberculosis: Recent advances and diagnostic algorithms. Tuberc. Respir. Dis..

[4] Lo C.-M., Wu Y.-H., Li Y.-C., Lee C.-C. (2020). Computer-aided bacillus detection in whole-slide pathological images using a deep convolutional neural network. Appl. Sci..

[5] Chang R.I., Chiu Y.H., Lin J.W. (2020). Two-stage classification of tuberculosis culture diagnosis using convolutional neural network with transfer learning. J. Supercomput..

[6] Swetha K., Sankaragomathi B., Thangamalar J.B. Convolutional neural network based automated detection of mycobacterium bacillus from sputum images. Proceedings of the 5th International Conference on Inventive Computation Technologies, ICICT 2020.

[7] Verma D., Bose C., Tufchi N., Pant K., Tripathi V., Thapliyal A. (2020). An efficient framework for identification of tuberculosis and pneumonia in chest x-ray images using neural network. Procedia Comput. Sci..

[8] Msonda P., Uymaz S.A., Karaağaç S.S. (2020). Spatial pyramid pooling in deep convolutional networks for automatic tuberculosis diagnosis. Trait. Signal.

[9] Singh J., Tripathy A., Garg P., Kumar A. (2020). Lung tuberculosis detection using anti-aliased convolutional networks. Procedia Comput. Sci..

[10] Bradley W.G. (2015). History of medical imaging. Proc. Am. Philos. Soc..

[11] Barani M., Mukhtar M., Rahdar A., Sargazi S., Pandey S., Kang M. (2021). Recent advances in nanotechnology-based diagnosis and treatments of human osteosarcoma. Biosensors.

[12] Douche D., Sert Y., Brandan S.A., Kawther A.A. (2021). 5-((1H-imidazol-1-yl) methyl) quinolin-8-ol as potential antiviral SARS-CoV-2 candidate: Synthesis, crystal structure, Hirshfeld surface analysis, DFT and molecular docking studies. J. Mol. Struct..

[13] Gümüş M., Babacan Ş.N., Demir Y., Sert Y., Koca İ., Gülçin İ. (2022). Discovery of sulfadrug–pyrrole conjugates as carbonic anhydrase and acetylcholinesterase inhibitors. Arch. Pharm..

[14] Murphy K., Habib S.S., Asad Zaidi S.M., Khowaja S., Khan A., Melendez J., Scholten E.T., Amad F., Schalekamp S., Verhagen M. (2020). Computer aided detection of tuberculosis on chest radiographs: An evaluation of the CAD4TB v6 system. Sci. Rep..

[15] Dou Q., Chen H., Yu L., Zhao L., Qin J., Wang D., Mok V.C.T., Shi L., Heng P.-A. (2016). Automatic detection of cerebral microbleeds from MR Images via 3D convolutional neural networks. IEEE Trans. Med. Imaging.

[16] Abbas A., Abdelsamea M.M., Gaber M.M. (2020). DeTrac: Transfer learning of class decomposed medical images in convolutional neural networks. IEEE Access.

[17] Zech J.R., Badgeley M.A., Liu M., Costa A.B., Titano J.J., Oermann E.K. (2018). Variable generalization performance of a deep learning model to detect pneumonia in chest radiographs: A cross-sectional study. PLoS Med..

[18] Shin H.C., Roberts K., Lu L., Demner-Fushman D., Yao J., Summers R.M. Learning to read chest x-rays: Recurrent neural cascade model for automated image annotation. Proceedings of the IEEE Computer Society Conference on Computer Vision and Pattern Recognition.

[19] Ronneberger O., Fischer P., Brox T. (2015). U-net: Convolutional networks for biomedical image segmentation. Lect. Notes Comput. Sci..

[20] Kotei E., Thirunavukarasu R. (2022). Computational techniques for the automated detection of mycobacterium tuberculosis from digitized sputum smear microscopic images: A systematic review. Prog. Biophys. Mol. Biol..

[21] Jaeger S., Candemir S., Antani S., Wáng Y.-X.J., Lu P.-X., Thoma G. (2014). Two public chest X-ray datasets for computer-aided screening of pulmonary diseases. Quant. Imaging Med. Surg..

[22] Stirenko S., Kochura Y., Alienin O., Rokovyj O., Gang P., Zeng W., Gordienko Y.G. Chest x-ray analysis of tuberculosis by deep learning with segmentation and augmentation. Proceedings of the 2018 IEEE 38th International Conference on Electronics and Nanotechnology, ELNANO 2018.

[23] Firmino M., Angelo G., Morais H., Dantas M.R., Valentim R. (2016). Computer-aided detection (CADe) and diagnosis (CADx) system for lung cancer with likelihood of malignancy. Biomed. Eng. Online.

[24] Brownlee J. Machine Learning Mastery. https://machinelearningmastery.com/what-is-deep-learning/.

[25] Fioravanti D., Giarratano Y., Maggio V., Agostinelli C., Chierici M., Jurman G., Furlanello C. (2018). Phylogenetic convolutional neural networks in metagenomics. BMC Bioinform..

[26] Grisoni F., Moret M., Lingwood R., Schneider G. (2020). Bidirectional molecule generation with recurrent neural networks. J. Chem. Inf. Model..

[27] Méndez-lucio O., Baillif B., Clevert D., Rouquié D., Wichard J. (2020). De novo generation of hit-like molecules from gene expression signatures using artificial intelligence. Nat. Commun..

[28] Su Y., Liu Q., Xie W., Hu P. (2022). YOLO-LOGO: A transformer-based YOLO segmentation model for breast mass detection and segmentation in digital mammograms. Comput. Methods Programs Biomed..

[29] Thai L.H., Hai T.S., Thuy N.T. (2012). Image Classification using Support Vector Machine and artificial neural network. Int. J. Inf. Technol. Comput. Sci..

[30] Pandit M.K., Banday S.A., Naaz R., Chishti M.A. (2020). Automatic detection of COVID-19 from chest radiographs using deep learning. Radiogr. J..

[31] Sitaula C., Hossain M.B. (2021). Attention-based VGG-16 model for COVID-19 chest X-ray image classification. Appl. Intell..

[32] Iqbal A., Latief J., Mudasir M. (2020). CoroNet: A deep neural network for detection and diagnosis of COVID-19 from chest x-ray images. Comput. Methods Programs Biomed..

[33] Ozturk T., Talo M., Yildirim E.A., Baloglu U.B., Yildirim O., Acharya U.R. (2020). Automated detection of COVID-19 cases using deep neural networks with X-ray images. Comput. Biol. Med..

[34] Showkatian E., Salehi M., Ghaffari H., Reiazi R., Sadighi N. (2022). Deep learning-based automatic detection of tuberculosis disease in chest X-ray images. Pol. J. Radiol..

[35] Priya E., Srinivasan S. (2016). Automated object and image level classification of TB images using support vector neural network classifier. Biocybern. Biomed. Eng..

[36] Ahmad T., Lund L.H., Rao P., Ghosh R., Warier P., Vaccaro B., Dahlstrom U., O’Connor C.M., Felker G.M., Desai N.R. (2018). Machine learning methods improve prognostication, identify clinically distinct phenotypes, and detect heterogeneity in response to therapy in a large cohort of heart failure patients. J. Am. Heart Assoc..

[37] Sankaran A., Jain A., Vashisth T., Vatsa M., Singh R. (2017). Adaptive latent fingerprint segmentation using feature selection and random decision forest classification. Inf. Fusion.

[38] Hooda R., Sofat S., Kaur S., Mittal A., Meriaudeau F. Deep-learning: A potential method for tuberculosis detection using chest radiography. Proceedings of the 2017 IEEE International Conference on Signal and Image Processing Applications, ICSIPA 2017.

[39] Hwang E.J., Park S., Jin K.-N., Kim J.I., Choi S.Y., Lee J.H., Goo J.M., Aum J., Yim J.-J., Parl C.M. (2019). Development and validation of a deep learning-based automatic detection algorithm for active pulmonary tuberculosis on chest radiographs. Clin. Infect. Dis..

[40] Devnath L., Luo S., Summons P., Wang D. (2018). Tuberculosis classification in chest radiographs using deep convolutional neural networks. Int. J. Adv. Sci. Eng. Technol..

[41] Cao Y., Liu C., Liu B., Brunette M.J., Zhang N., Sun T., Zhang P., Peinado J., Garavito E.S., Garcia L.L. Improving tuberculosis diagnostics using deep learning and mobile health technologies among resource-poor and marginalized communities. Proceedings of the 2016 IEEE 1st International Conference on Connected Health: Applications, Systems and Engineering Technologies, CHASE 2016.

[42] Liu C., Cao Y., Alcantara M., Liu B., Brunette M.J., Peinado J., Curioso W. TX-CNN: Detecting tuberculosis in chest x-ray images using convolutional neural network. Proceedings of the International Conference on Image Processing, ICIP.

[43] Heo S.J., Kim Y., Yun S., Lim S.-S., Kim J., Nam C.-M., Park E.-C., Jung I., Yoon J.-H. (2019). Deep learning algorithms with demographic information help to detect tuberculosis in chest radiographs in annual workers’ health examination data. Int. J. Environ. Res. Public Health.

[44] Hwang E.J., Park S., Jin K.-N., Kim J.I., Choi S.Y., Lee J.H., Goo J.M., Aum J., Yim J.-J., Cohen J.G. (2019). Development and validation of a deep learning-based automated detection algorithm for major thoracic diseases on chest radiograph. JAMA Netw. Open.

[45] Gozes O., Greenspan H. Deep feature learning from a hospital-scale chest x-ray dataset with application to TB detection on a small-scale dataset. Proceedings of the Annual International Conference of the IEEE Engineering in Medicine and Biology Society, EMBS.

[46] Pasa F., Golkov V., Pfeiffer F., Cremers D., Pfeiffer D. (2019). Efficient deep network architectures for fast chest x-ray tuberculosis screening and visualization. Sci. Rep..

[47] Rohilla A., Hooda R., Mittal A. (2017). TB detection in chest radiograph using deep learning architecture. Int. J. Adv. Res. Sci. Eng..

[48] Yadav O., Passi K., Jain C.K. Using Deep learning to classify x-ray Images of potential tuberculosis patients. Proceedings of the 2018 IEEE International Conference on Bioinformatics and Biomedicine, BIBM 2018.

[49] Akbar S., GhaniHaider N., Tariq H. (2019). Tuberculosis diagnosis using x-ray images. Int. J. Adv. Res..

[50] Devnath L., Luo S., Summons P., Wang D. (2021). Automated detection of pneumoconiosis with multilevel deep features learned from chest X-ray radiographs. Comput. Biol. Med..

[51] Rajpurkar P., Irvin J., Zhu K., Yang B., Mehta H., Duan T., Ding D., Bagul A., Langlotz C., Shpanskaya K. (2017). CheXNet: Radiologist-Level Pneumonia Detection on Chest X-rays with Deep Learning. arXiv.

[52] CDC (Center for Diseases Control and Prevention). https://www.cdc.gov/niosh/topics/cwhsp/cwhsp-xray.html.

[53] Rahman T., Khandakar A., Abdul Kadir M., Islam K.R., Islam K.F., Mazhar R., Hamid T., Islam M.T., Kashem S., Mahbub Z.B. (2020). Reliable tuberculosis detection using chest x-ray with deep learning, segmentation and visualization. IEEE Access.

[54] He K., Zhang X., Ren S., Sun J. Deep residual learning for image recognition. Proceedings of the IEEE Computer Society Conference on Computer Vision and Pattern Recognition.

[55] Szegedy C., Vanhoucke V., Ioffe S., Shlens J., Wojna Z. (2016). Rethinking the inception architecture for computer vision. Proc. IEEE Comput. Soc. Conf. Comput. Vis. Pattern Recognit..

[56] Simonyan K., Zisserman A. Very deep convolutional networks for large-scale image recognition. Proceedings of the 3rd International Conference on Learning Representations, ICLR 2015—Conference Track Proceedings.

[57] Huang G., Liu Z., van der Maaten L., Weinberger K.Q. Densely connected convolutional networks. Proceedings of the 30th IEEE Computer Vision and Pattern Recognition, CVPR.

[58] Iandola F.N., Han S., Moskewicz M.W., Ashraf K., Dally W.J., Keutzer K. (2016). SqueezeNet: AlexNet-level accuracy with 50× fewer parameters and <0.5 MB model size. arXiv.

[59] Howard A.G., Zhu M., Chen B., Kalenichenko D., Wang W., Weyand T., Andreetto M., Adam H. (2017). MobileNets: Efficient Convolutional Neural Networks for Mobile Vision Applications. arXiv.

[60] Guo R., Passi K., Jain C.K. (2020). Tuberculosis diagnostics and localization in chest x-rays via deep learning models. Front. Artif. Intell..

[61] Abideen Z.U., Ghafoor M., Munir K., Saqib M., Ullah A., Zia T., Tariq S.A., Ahmed G., Zahra A. (2020). Uncertainty assisted robust tuberculosis identification with bayesian convolutional neural networks. IEEE Access.

[62] Li X., Zhou Y., Du P., Lang G., Xu M., Wu W. (2020). A deep learning system that generates quantitative CT reports for diagnosing pulmonary Tuberculosis. Appl. Intell..

[63] Sahlol A.T., Elaziz M.A., Jamal A.T., Damaševičius R., Hassan O.F. (2020). A novel method for detection of tuberculosis in chest radiographs using artificial ecosystem-based optimisation of deep neural network features. Symmetry.

[64] Munadi K., Muchtar K., Maulina N., Pradhan B. (2020). Image enhancement for tuberculosis detection using deep learning. IEEE Access.

[65] Rajaraman S., Candemir S., Xue Z., Alderson P.O., Kohli M., Abuya J., Thoma G.R., Antani S. A novel stacked generalization of models for improved TB detection in chest radiographs. Proceedings of the Annual International Conference of the IEEE Engineering in Medicine and Biology Society, EMBS.

[66] Lopes U.K., Valiati J.F. (2017). Pre-trained convolutional neural networks as feature extractors for tuberculosis detection. Comput. Biol. Med..

[67] Ayaz M., Shaukat F., Raja G. (2021). Ensemble learning based automatic detection of tuberculosis in chest X-ray images using hybrid feature descriptors. Phys. Eng. Sci. Med..

[68] Lu P.X. Chest X-ray Masks and Label. Kaggle. https://www.kaggle.com/nikhilpandey360/chest-xray-masks-and-labels.

[69] Kaggle RSNA Pneumonia Detection Challenge. [Online]. https://www.kaggle.com/c/rsna-pneumonia-detection-challenge/data.

[70] Kaggle Tuberculosis (TB) Chest X-ray Database. https://www.kaggle.com/datasets/tawsifurrahman/tuberculosis-tb-chest-xray-dataset.

